# Supplementary material for: Tunneling anisotropic magnetoresistance driven by magnetic phase transition
Source: Nat Commun. 2017 Sep 6;8:449. doi: 10.1038/s41467-017-00290-4 (PMC5587625; doi:10.1038/s41467-017-00290-4)
Supplement: Supplementary file 1 — Supplementary Information [file 41467_2017_290_MOESM1_ESM.pdf]

File Name: Supplementary Information
Description: Supplementary Figures, Supplementary Notes and Supplementary References.

File Name: Peer Review File
Description:

Supplementary Note 1. Comparison of magnetization phase transition of FeRh and FeRh/MgO

Supplementary Figure 1 illustrates the temperature-dependent magnetic phase transition of 30 nm-thick α'-FeRh and α'-FeRh capped with MgO. The magnetic phase transition temperature of α'-FeRh capped with MgO is lower than that of α'-FeRh single layer, which is supported by the previous reports[1, 2]. Pan *et. al.* [1] probed the Rh-rich FeRh near the FeRh/MgO interface, where the Rh-rich (Fe-deficient) composition resulted in the decrease of the phase-transition temperature[2]. Also visible in Supplementary Fig. 1 is that the heterogeneity of FeRh with the MgO capping layer widens the hysteresis of magnetic phase transition. Since the magnetic layer in the vicinity of the tunneling barrier dominates the tunneling effect, the magnetic phase transition tunneling anisotropic magnetoresistance (PT-TAMR), sensitive to the magnetic phase transition of interfacial α'-FeRh instead of that of the bulk α'-FeRh, shows the lower transition temperature and wider transition hysteresis in Fig. 2b, compared to the scenario of the bulk α'-FeRh (Fig. 2a).

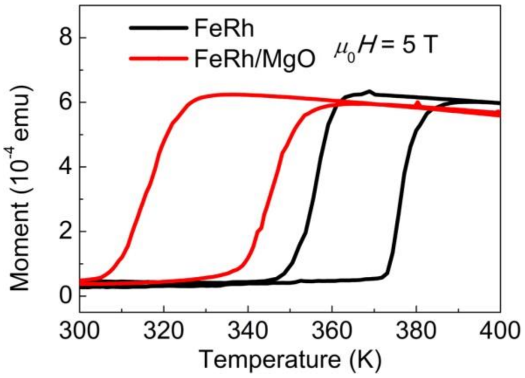

Supplementary Figure 1 | The impact of MgO capping on the magnetic phase transition of α'-FeRh. Magnetic moment versus temperature curves for α'-FeRh and α'-FeRh/MgO bilayers at 5 Tesla are shown.

Supplementary Note 2. Endurance of the PT-TAMR effect

The strain effect induced by the magnetic phase transition might result in the degradation of the PT-TAMR effect. Nevertheless the lattice is almost expanded in the *c*-axis direction, while the in-plane lattice of α'-FeRh keeps almost constancy due to the clamping effect from MgO substrate[3]. Therefore the structure of tunneling interface (α'-FeRh/MgO) should suffer little from the lattice expansion induced by the magnetic phase transition. Supplementary Figure 2 shows the magnetic field dependent resistance (RA–μ_0H) at 300 K in ten circles, which keeps almost unchanged, indicating good endurance of the present devices in ten circles.

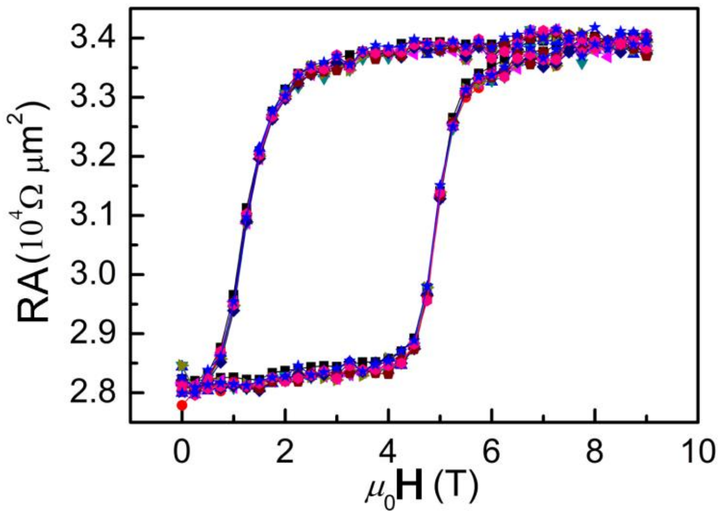

Supplementary Figure 2| **Magnetic field dependent resistance (RA–μ_0H) at 300 K in ten circles.** Magnetic field were scanned from 0 to 9 Tesla to trigger the magnetic phase transitions in ten circles.

Supplementary Note 3. Influence of spin-orbit coupling on DOS of α'-FeRh

Considering that spin-orbit coupling (SOC) is crucial for the previous TAMR effect[4], we also performed DOS calculation of α'-FeRh with SOC at the

α'-FeRh/MgO interface. It is found that the DOS variation induced by SOC is negligible when comparing with the DOS difference at AFM and FM states. Thus it is concluded that the origin of the present TAMR is not related to SOC but driven by magnetic phase transition and the resultant change of electronic structure.

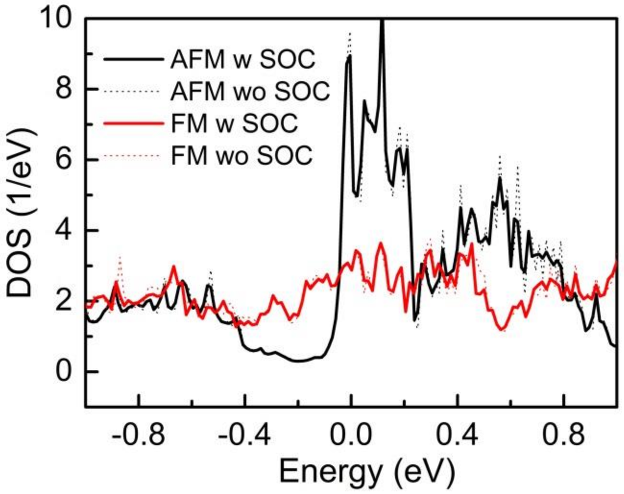

Supplementary Figure 3 | DOS comparison of α'-FeRh at the α'-FeRh/MgO interface with and without spin-orbit coupling. The DOS of α'-FeRh at AFM and FM states with (w) and without (wo) spin-orbit coupling are shown by the solid line and dotted line, respectively.

Supplementary Note 4. MgO/γ-FeRh top interface of α'-FeRh/MgO/γ-FeRh junctions

Supplementary Figure 4a presents a high resolution scanning transmission electron microscopy (STEM) Z-contrast image of the MgO/γ-FeRh top interface. Although some defects exist in the γ-FeRh due to the lattice mismatch, the MgO/γ-FeRh interface is sharp and keeps epitaxial growth mode to some extent, which is the main motivation for us to employ non-magnetic γ-FeRh as the top electrode. We show in Fig. S4b the electron energy loss spectra (EELS) of Fe close to

the MgO/γ-FeRh interface. The technique is identical to the one used for the α'-FeRh/MgO bottom interface. Three EELS curves were recorded, which are denoted as 1, 2, and 3, with the order of increasing the distance from the interface. The space gap for the curves is ~0.4 nm with respect to the lattice parameter of γ-FeRh. Remarkably, all of the EELS data exhibit identical energy position within the sensitivity of our EELS, indicating the existence of Fe^0 at the MgO/γ-FeRh top interface and negligible oxidation. This is different from the scenario of the α'-FeRh/MgO interface. This difference could be explained by two effects: i) because of different surface energy of MgO and FeRh, oxygen is easier pumped into FeRh when MgO is deposited by magnetron sputtering, whereas oxygen diffusion is less when FeRh is grown on MgO. A similar phenomenon was reported in Fe/MgO/Fe stacks[5]; ii) The dense fcc structure of γ-FeRh probably serve as the obstacles for oxygen diffusion.

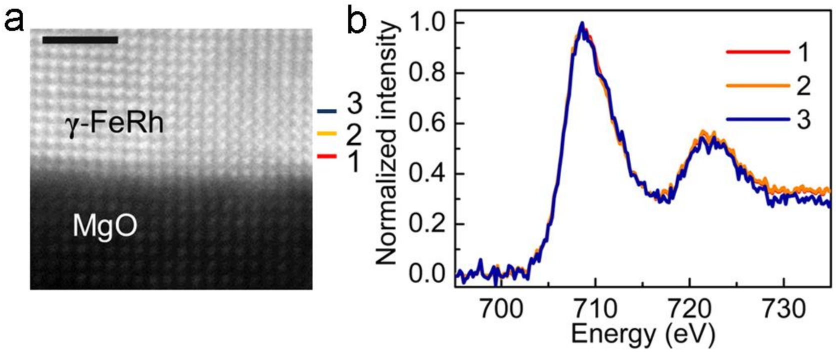

Supplementary Figure 4| STEM and EELS characterization of MgO/γ-FeRh top interface. a, high resolution STEM Z-contrast image of the MgO/γ-FeRh top interface. Scale bar is 1 nm in length. **b**. Fe $L_{2,3}$-edge EELS marked as 1, 2, and 3 in the order of increasing the distance from the MgO/γ-FeRh interface with ~0.4 nm gap.

Supplementary Note 5. Enhancement of DOS at AFM state due to Fe-O

hybridization

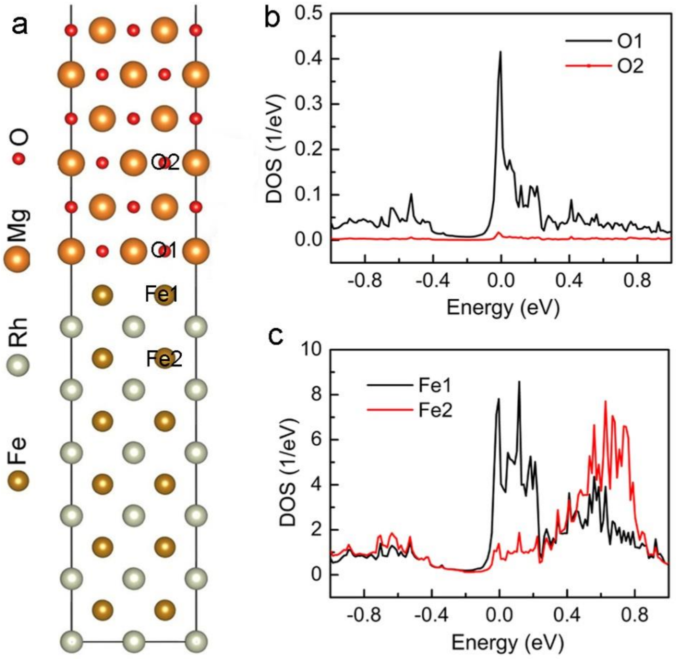

Supplementary Figure 5 | DOS for the Fe and O atoms in the vicinity of the α'-FeRh/MgO interface when α'-FeRh is at AFM state. a, α'-FeRh/MgO supercell used for DOS calculation. b, Comparison of DOS for O1 and O2 atoms, in which O1 is at the first neighbor of the α'-FeRh/MgO interface and O2 is one unit cell away from O1. c, Comparison of DOS for Fe1 and Fe2 atoms, in which Fe1 is at the first neighbor of the α'-FeRh/MgO interface and Fe2 is one unit cell away from Fe1.

Supplementary Figure 5 illustrates the α'-FeRh/MgO supercell and corresponding DOS for the Fe and O atoms in the vicinity of the α'-FeRh/MgO interface when α'-FeRh is at AFM state. The most eminent feature in the figure is the enhanced DOS at the Fermi level for both Fe1 and O1 (Fig. S5b and S5c), which locates the first neighbor of the α'-FeRh/MgO interface, as marked in Fig. S5a. However, such a feature vanishes for Fe2 and O2, which is one unit cell away from Fe1 and O1, respectively. This comparison demonstrates that Fe-O hybridization plays a profound

role on the enhancement of DOS of Fe for AFM α’-FeRh. Such a high DOS for AFM α’-FeRh leads to the low tunneling resistance of AFM α’-FeRh and the resultant positive PT-TAMR of α’-FeRh-based junctions, as shown in Fig. 3. On the other hand, the PT-TAMR effect is so sensitive to the α’-FeRh/MgO interface that both the polarity and amplitude of the PT-TAMR could be strongly manipulated by the design of α’-FeRh/MgO interface.

Supplementary Note 6. Transmission distribution in two dimensional Brillouin zone of majority-spin channels

As shown in Supplementary Fig. 6, the transmission of majority-spin channels for both α’-FeRh/MgO(2.5 u.c.)/Cu and α’-FeRh/Rh(1 u.c.)/MgO(2.5 u.c.)/Cu junctions is lower compared with that of their minority-spin channels (Fig. 4), indicating the negligible role on tunneling conductance. The transmission of both majority-spin and minority-spin channels (Fig. 4a) at AFM state for α’-FeRh/MgO/Cu are identical taking the spin degeneracy into account. Therefore, only the transmission at FM state is shown in Supplementary Fig. 6a. Given that the induced magnetic moment in interfacial 1 u.c. fcc-Rh breaks the spin degeneracy, besides the FM case, the transmission of majority channels at AFM state for α’-FeRh/Rh/MgO/Cu junctions is also presented in Supplementary Fig. 6b. Although the transmission at the AFM (Supplementary Fig. 6b) is stronger than that at FM state (Supplementary Fig. 6c), both of them are much weaker than their minority counterparts. Thus the tunneling resistance is still determined by the transmission of minority-spin channels (Fig. 4c and d), giving rise to the negative polarity of PT-TAMR in this case.

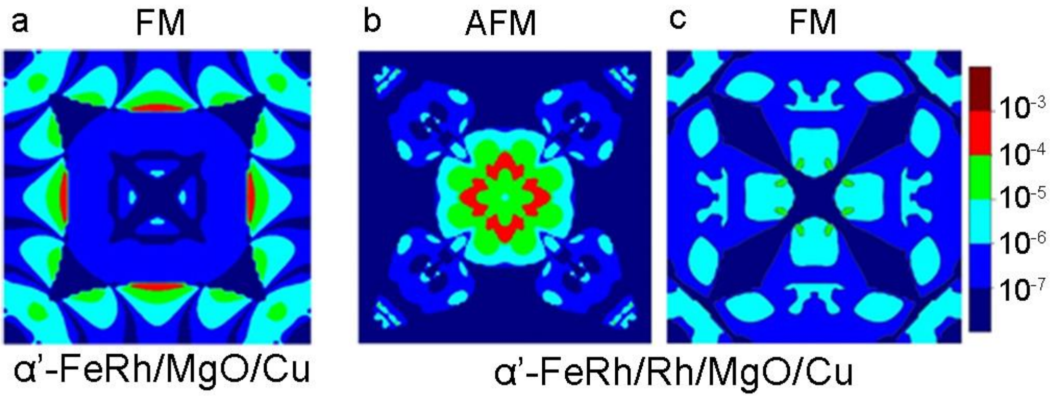

Supplementary Figure 6 | Transmission distribution in Two dimensional Brillouin zone for α'-FeRh/MgO(2.5 u.c.)/Cu and α'-FeRh/Rh(1 u.c.)/MgO(2.5 u.c.)/Cu junctions at Fermi level. a, majority-spin channels at FM state for α'-FeRh/MgO/Cu junctions. b,c, majority-spin channels at AFM and FM states for α'-FeRh/Rh/MgO/Cu junctions, respectively.

Supplementary Note 7. Temperature- and bias-dependent tunneling behavior of the annealed α'-FeRh/MgO/γ-FeRh junctions

Supplementary Figure 7a shows the temperature-dependent Resistance-area (RA–*T*) product of the annealed α'-FeRh/MgO/γ-FeRh junctions with three typical magnetic fields. The most striking result is the transition from high resistance state (HRS) to low resistance state (LRS), indicating the negative polarity, in contrast to the case of the junctions without annealing. In the temperature region away from the magnetic phase transition, the RA drops with increasing temperature, reaffirming the tunneling behavior of the annealed junction. In addition, the temperature for the magnetic phase transition decreases with enhancing the magnetic field from 1 to 7 T, coinciding with their counterparts without annealing in Fig. 2b. The bias dependence of the room temperature RA at 0 T and 9 T is shown in Supplementary Fig. 7b. Remarkably, the RA at 0 T in the bias region shown is higher than that at 9 T,

confirming the HRS and LRS at the antiferromagnetic (at 0 T) and ferromagnetic state (at 9 T), respectively.

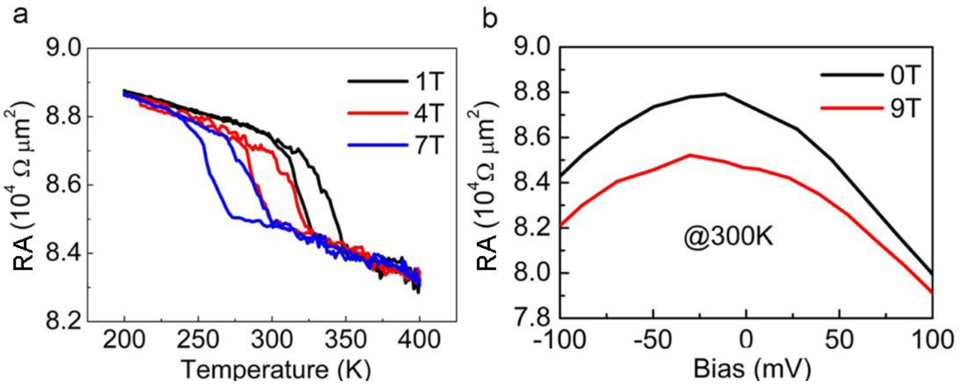

Supplementary Figure 7 | Temperature and bias dependent RA. a, RA versus temperature with magnetic fields of 1, 4 and 7 T for the annealed junction. **b**, Bias dependent RA at 0 T and 9 T.

Supplementary Note 8. Thermal assisted nonvolatile functionality

Given that the magnetic phase transition of FeRh is the first order phase transition with a hysteresis window, the PT-TAMR effect naturally shows a nonvolatile memory function. Supplementary Figure 8 displays that two different resistance states of α'-FeRh/MgO/γ-FeRh junctions could be preserved at zero-field after superheating and undercooling processes. For this measurement, superheating was carried out with the temperature increasing to 400 K followed by cooling back to 330 K, a temperature almost locating at the middle of the hysteresis window for the AFM-FM transition at zero-field. In this case, α'-FeRh keeps at FM state and a higher RA for 30 min (the black line in Supplementary Fig. 8). Similarly, when the junction was cooled down to 250 K and then warmed back to 330 K, it stays at AFM state accompanied by a stable low resistance for 30 min (the red line in Supplementary Fig. 8). Three circles of such measurements were carried out to confirm that the nonvolatile function of the

PT-TAMR effect is reproducible.

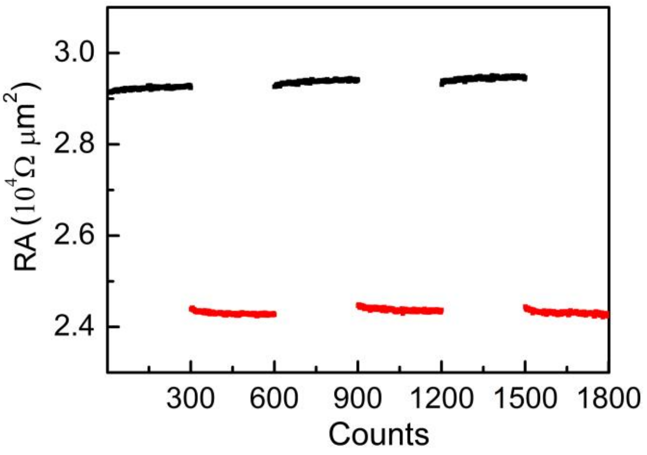

Supplementary Figure 8 | Resistance area (RA) recorded at 330 K and zero-field after superheating and undercooling. α'-FeRh at FM and AFM states after superheating and undercooling correspond to high (black line) and low resistances (red lines) of α'-FeRh/MgO/γ-FeRh junctions, respectively. Each line is composed by 300 counts recorded in 30 min. Three circles of such measurements are shown in the figure.

Supplementary References

1. Fan, R. *et al.* Ferromagnetism at the interfaces of antiferromagnetic FeRh epilayers. *Phys. Rev. B* **82**,184418 (2010).

2. Van Driel. J., Coehoorn, R., Strijkers, G. J., Brück, E. & de Boer, F. R. Compositional dependence of the giant magnetoresistance in Fe_xRh_{1-x} thin films. *J. Appl. Phys.* **85,** 1026–1036 (1999).

3. Bordel, C. *et al.* Fe spin reorientation across the metamagnetic transition in strained FeRh thin films. *Phys. Rev. Lett.* **109**, 117201 (2012).

4. Park, B. G. *et al.* Tunneling anisotropic magnetoresistance in multilayer (Co/Pt)/AlO_x/Pt structures. *Phys. Rev. Lett.* **100**, 087204 (2008).

5. Tusche, C. *et al*. Oxygen-induced symmetrization and structural coherency in Fe/MgO/Fe (001) magnetic tunnel junctions. *Phys. Rev. Lett.* **95**, 176101 (2005).
